# Monocyte Function in the Fetus and the Preterm Neonate: Immaturity Combined with Functional Impairment

**DOI:** 10.1155/2013/753752

**Published:** 2013-04-09

**Authors:** Zoe Iliodromiti, Anastasis Anastasiadis, Michail Varras, Kalliopi I. Pappa, Charalambos Siristatidis, Vassilios Bakoulas, George Mastorakos, Nikolaos Vrachnis

**Affiliations:** ^1^Neonatal Unit, 2nd Department of Obstetrics and Gynecology, University of Athens Medical School, Aretaieio Hospital, 11528 Athens, Greece; ^2^Department of Obstetrics and Gynecology, General District Hospital “Helena Venizelou”, 11521 Athens, Greece; ^3^1st Department of Obstetrics and Gynecology, University of Athens Medical School, “Alexandra” Hospital, 11528 Athens, Greece; ^4^3rd Department of Obstetrics and Gynecology, University of Athens Medical School, Attikon Hospital, Haidari, 12462 Attiki, Greece; ^5^2nd Department of Obstetrics and Gynecology, University of Athens Medical School, Aretaieio Hospital, 11528 Athens, Greece

## Abstract

It is well known that the innate immunity system, involving the contribution of monocytes and macrophages, may dysfunction in fetuses and preterm neonates. Monocytes are capable of differentiating into dendritic cells (DCs) or into mucosal macrophages during certain infections and of producing inflammatory mediators such as TNF-**α** (tumor necrosis factor-alpha), nitric oxide, and reactive oxygen species. Fetuses as well as neonates are prone to infections as a result of a defective mechanism within the above mononuclear system. Monocyte function in fetuses and preterm neonates depends on the phagocytic and oxidative capacity of macrophages and their antigen-adhesion ability. Functional rather than anatomical impairment is probably the underlying cause, while a defective production of cytokines, such as TNF-**α**, IL-6 (Interleukin 6), IL-1**β** (Interleukin 1 beta), and G-CSF (Granulocyte Colony-Stimulating Factor), has also been involved. The insufficient production of the above inflammatory mediators and the phenomenon of endotoxin intolerance, which latter occurs during entry of any antigen into the premature neonate, place preterm neonates at higher risk for infections. Existing research data are herein presented which, however, are deficient and fragmental, this accounting for the fact that the precise pathophysiology of these disturbances is not yet fully clarified.

## 1. Introduction

Monocytes and macrophages, which are categorized among the leukocytes, play a pivotal role in the innate immune system [1–3]. Their origin lies in a common myeloid precursor, while they both play important roles in innate immunity. Macrophages, which are produced from monocytes after their exposure to certain stimuli, circulate in the blood stream; they are short-lived as they undergo spontaneous apoptosis [4, 5]. However, in response to differentiating factors, some monocytes migrate and populate tissues, thereby escaping their apoptotic fate and become macrophages which have a longer lifespan and are found in almost every organ of the body. Residing as phagocytic cells in lymphoid and nonlymphoid tissue, they have the ability to recognize a broad range of pathogens and are efficient phagocytes, while they also induce the production of inflammatory cytokines [6–8].

Monocytes have the potential to differentiate to dendritic cells (DCs) or tissue macrophages, although many DCs and tissue macrophages do not originate from monocytes in a steady state [4]. Under certain circumstances, monocytes do differentiate into DCs during infection that produce inflammatory mediators such as TNF-*α*, nitric oxide (NO^−^) and reactive oxygen species, as in *Listeria monocytogenes* infection [7, 9]. Nevertheless, under other conditions, monocytes differentiate into mucosal macrophages with a different surface phenotype and capability of inflammatory mediators production, as happens in *Toxoplasma gondii* infection [10]. Monocytes and their precursors can either activate or inhibit immune response, depending on local and systemic signals and the pathogen involved [4].

 Monocytes acquire capability of differentiation and inflammatory mediators production early in life [7, 11]. Nevertheless, there is evidence of immaturity of the innate immune system in human fetuses, including its fundamental component, the mononuclear phagocyte system. This immaturity is thought to be functional in origin, as the various cell types involved in innate immunity are present in the fetus, although their immunity “virtues” are in question [12]. Neonates are prone to severe bacterial infections, and the mortality caused by infections is high despite prompt antibiotic therapy: this phenomenon is especially prevalent among preterm neonates [13]. As production of phagocytes and their proper functioning are crucial for an effective bactericidal immune mechanism, defective functioning of the mononuclear system may account for the vulnerability of neonates to infections [14].

## 2. Monocyte Function in Fetuses and Preterm Neonates

Few studies on the fetus and neonate immune system are available and knowledge is sparse as regards the phagocytic and oxidative capacity of macrophages or their adhesion molecule expression [15–19]. Nevertheless, it has been determined that various functions of the fetal innate immune system are essentially different from those observed in term neonates or adults [11, 20]. A considerably diminished phagocytic activity of fetal monocytes has been described, this manifesting in sharp contrast to elevated production of reactive oxygen products. Moreover, significant numbers of fetal monocytes are capable of production of proinflammatory cytokines in response to inflammatory stimulation [11]. A difference, however, was noted in the pattern of cytokine production as compared with that demonstrated in more mature individuals; in other words, there was notable diminishment in the number of IL-6 and tumor necrosis factor-positive monocytes. In contrast to term infant responses, TNF-*α* and IL-6 responses of preterm infants (with a gestation age (GA) of <30 weeks) were severely impaired due to a diminished proportion of cytokine producing monocytes. Likewise, responses among preterm monocytes with respect to whole cord blood stimulated with live Group B Streptococci (GBS) were found to be almost invariably less robust than those noted in term newborns [12]. 

In neonates, the expression of several adhesion molecules involved in monocyte migration has been found to be appreciably compromised, thereby conducing to a diminishment in neonatal immune response. Moreover, qualitative deficiencies of phagocyte function have been demonstrated in preterm neonates [21]. The fact that newborn infants, particularly those delivered prematurely, generate comparatively low quantities of G-CSF after inflammatory stimulation suggests that this may partially account for their deficient upregulation of both neutrophil production and function during infection [22].

**Figure 1:**
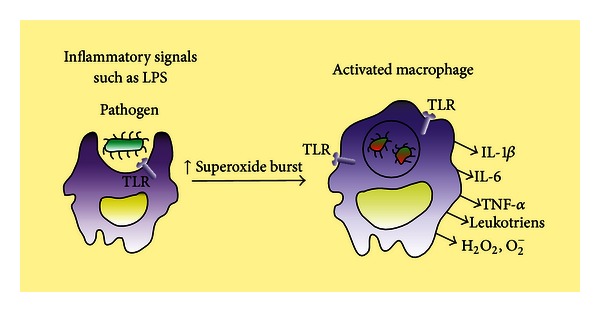
The intrusion of bacteria stimulates the tissue macrophages, which produce IL-1*β*, IL-6, and TNF-*α*. While the macrophages undergo activation via exposure to TLR agonists, such as lipopolysaccharide (LPS), the superoxide burst is increased through microbial killing in activated macrophages. This intrusion stimulates the tissue macrophages which produce cytokines (IL-1*β*, IL-6, TNF-*α*, etc.), reactive oxygen species (e.g., H_2_O_2_ and superoxide anion), and leukotrienes.

As regards the impact of prematurity on monocyte phagocytosis, data are very limited. Nevertheless, investigations into preterm neutrophils and preterm rabbit alveolar macrophages have documented reduced bacterial uptake [23, 24], while other bacterium-derived stimuli have indicated that monocytes from preterm neonates possess an intrinsic gestation age-related deficiency in their ability to recognize and respond to GBS [12, 25]. Meanwhile, it is well established that decreased TNF-*α* secretion, bactericidal function, and adherence receptor expression in monocytes occur in preterm neonates [7, 11, 12] (Figure 1).

The decreased monocyte function could possibly explain the equivalence of DNA content between preterm and full-term monocytes as demonstrated via DNA analysis [12, 21]. Apart from DNA content, equivalence was also shown between secretion of the cytokines IL-1*β* and IL-6 by adherent monocytes in both preterm and term neonates. The same phenomenon is observed with regard to superoxide anion (O_2_^−^) production and degranulation, which are equivalent or elevated in freshly isolated monocytes from preterm neonates compared to full-term neonates. The above observations strongly indicate the presence and full functionality of the essential cellular constituents of the bactericidal response in monocytes from preterm neonates [21]. Moreover, monocytes placed in culture show that the processes of adherence and differentiation bring about changes in protein synthesis and cellular metabolism, by which modifications in turn modulate bactericidal activity. Thus, the decreased O_2_^−^ production and degranulation observed in preterm adherent monocytes appear to be related to regulatory processes rather than defective or absent components of the bactericidal response [21]. On the other hand, one may also hypothesize that slight modifications take place in the adherence capacity of preterm neonates' monocytes, which would exert effects on signaling pathways regulating TNF-*α* secretion. It is of note in this regard that production of IL-1*β* and IL-6 was seen to be unchanged in preterm cells, this suggesting the functioning of regulatory controls different from those of TNF-*α* [12].

## 3. Deficiency in Monocytes of the Preterm Neonates: Functional, Structural, or Both?

Two main research questions have been posed: is it an anatomy issue, that is, is there a structural defect in preterm monocytes, or does it concern a functional impairment? The few available observations in preterm infancy tend to point to the second explanation without furnishing clear-cut elucidation. Findings regarding integrity of monocyte phagocytic mechanism indicate the presence and total functionality of the essential cellular components of the bactericidal activity in monocytes from preterm newborns. Diminished cell count, if present, may conceivably account for the decreased monocyte function. Consequently, anatomical impairment may be excluded and functional impairment, at least an intrinsic one, is under question. When the secretion of IL-1*β* is examined, IL-1*β* response to lipopolysaccharide (LPS) seems to be intact in newborn human monocytes, while an increased unstimulated activity of these following neonatal complications has also been recorded [18].

Assuming that the deficiency in monocytes of the preterm neonates may be an anatomy issue of the innate immune system, this could come about as a result of allelic mutations at a single locus Toll-like receptor 4 (TLR4) gene, since this is the only pathway serving LPS. It is noteworthy in this respect that these mutations of the TLR4 gene are responsible for endotoxin resistance. The ability of the host to respond to antigens alters as the gene-sequence changes, which means that the differences in LPS response in humans are associated with common mutations in the particular gene, this effect also being corroborated by genetic evidence [26, 27]. Furthermore, in some populations of high-risk preterm infants, TLR4 genotypes may influence the BPD (biparietal diameter of the fetus) severity [28].

Regarding IL-6, data are somewhat conflicting, since equivalent correspondence between secretion of the cytokines IL-1*β* and IL-6 by adherent monocytes in preterm, on the one hand, and term neonates, on the other hand, has been determined [17]. Nevertheless, the induced IL-6 levels were significantly low in preterm neonates, especially in those born before 30 weeks of gestation. Neither the low serum immunoglobulin G levels accompanied by low serum complement levels in preterm neonates nor the slightly reduced monocytes number provided a sufficient explanation, since the level of IL-6 induced by LPS (LPS stimulation presumably does not require the opsonization process) remained lower when the newborns were less than 30 weeks of gestational age [12]. However, differences in monocyte numbers alone are not large enough to explain the marked differences in IL-6 levels produced by preterm and term neonates. One may thus speculate that preterm neonates are inherently defective in monocyte functions [12]. The synthesis of IL-6 and G-CSF among other cytokines in preterm and term newborns in an ex vivo cord blood culture endotoxin model opsonization appears to be related to immaturity [29]. 

The suppressed production of IL-6, along with diminished production of hydrogen peroxide, is considered typical of the so-called endotoxin intolerance [30]. The neonate cells are capable of responding appropriately to the antigen presentation and the direct microbicidal activity and oxidative burst. In this context, normally lethal doses of endotoxin can be tolerated if the organism has been previously exposed to sublethal doses of endotoxin. In the clinical setting, this state of immune tolerance renders the organism vulnerable to nosocomial infections: presentation in neonates of indolent, and persistent chorioamnionitis most probably is a manifestation of this high-risk condition [13, 31]. 

The published studies to date have some limitations, partly because of the inherent difficulties of studying a crucial part of the immune system separately from the whole system. In this context, the use of isolated mononuclear cells often results in the nonspecific stimulation of these cells to produce various cytokines. On the other hand, it has become evident that an ideal platform for assessment of the cellular response to stimulating agents in vitro may be provided via cultures using whole blood or human placenta and myometrium and examining their interactions with the immune system during labor [32]. The difficulties in studying the innate immune system of human neonates also stems from the fact that release of cytokines and inflammatory mediators may occur in other nonpathological situations as well, such as pregnancy and labor, and not merely in infections [32]. In sum, the above findings, established in humans, strongly indicate that the same could also arise in preterm neonates, further studies being required to investigate this hypothesis.

However, in vitro studies may not adequately describe in vivo processes, since monocytes in vivo act as part of the whole immune system, under real conditions. Moreover, multiple level interactions with other components of the immune system do happen and in vitro studies may be considered as an oversimplification.

## 4. Conclusions

In conclusion, although it is well established that monocytes have a central role in innate immunity and their defective function in preterm neonates is recognized, there is as yet scant knowledge about the pathophysiological mechanisms that account for this functional impairment. The greatest degree of impairment is most likely to be encountered in those neonates that were born at around 30 weeks of gestational age. This defective response to pathogens along with endotoxin intolerance may well place neonates at risk for nosocomial infection. Future research should focus on production of specific molecules, such as cytokines and other mediators of inflammation, along with their interaction with other components of the immune system. Via precise identification of the mechanism of deficient immune system response in preterm neonates, it may be possible in the future to successfully address infection in these patients by achieving speedy activation of innate immune mechanisms as well as, if feasible, acceleration of monocyte maturation.
